# Bridging the Technological Divide: Stigmas and Challenges With Technology in Digital Brain Health Studies of Older Adults

**DOI:** 10.3389/fdgth.2022.880055

**Published:** 2022-04-29

**Authors:** Jessica Nicosia, Andrew J. Aschenbrenner, Sarah L. Adams, Marisol Tahan, Sarah H. Stout, Hannah Wilks, Joyce E. Balls-Berry, John C. Morris, Jason Hassenstab

**Affiliations:** ^1^Charles F. and Joanne Knight Alzheimer's Disease Research Center, Department of Neurology, Washington University School of Medicine, St. Louis, MO, United States; ^2^Department of Neurology, Washington University School of Medicine, St. Louis, MO, United States; ^3^Department of Psychological and Brain Sciences, Washington University in St. Louis, St. Louis, MO, United States

**Keywords:** aging, smartphones, remote cognitive assessment, Alzheimer's disease, technology

## Abstract

The COVID-19 pandemic has increased adoption of remote assessments in clinical research. However, longstanding stereotypes persist regarding older adults' technology familiarity and their willingness to participate in technology-enabled remote studies. We examined the validity of these stereotypes using a novel technology familiarity assessment (*n* = 342) and with a critical evaluation of participation factors from an intensive smartphone study of cognition in older adults (*n* = 445). The technology assessment revealed that older age was strongly associated with less technology familiarity, less frequent engagement with technology, and higher difficulty ratings. Despite this, the majority (86.5%) of older adults elected to participate in the smartphone study and showed exceptional adherence (85.7%). Furthermore, among those enrolled, neither technology familiarity, knowledge, perceived difficulty, nor gender, race, or education were associated with adherence. These results suggest that while older adults remain significantly less familiar with technology than younger generations, with thoughtful study planning that emphasizes participant support and user-centered design, they are willing and capable participants in technology-enabled studies. And once enrolled, they are remarkably adherent.

## Introduction

At the emergence of the World Wide Web, the internet was only accessible to wealthy, technologically savvy people in Western, educated, industrialized, rich, and democratic (WEIRD) societies. Today, it connects billions of people around the world and, especially due to the increased availability and accessibility of smartphones, it has become ingrained in our everyday lives.

What does this mean for clinical studies of brain health, and specifically those of Alzheimer's disease (AD)? Before the SARS-CoV-2 (COVID-19) pandemic, researchers had begun to adopt remote platforms (i.e., Amazon Mechanical Turk, Prolific) to acquire larger, more generalizable samples [(1, 2) however, see also (3)]. The COVID-19 pandemic forced studies to rapidly transition to remote testing methods to continue data collection, which has drawbacks, but also many well-understood advantages over laboratory-based studies (4). Remote studies allow for increased efficiency of data collection (5) such that recruitment and testing can happen simultaneously for many studies and participants. Converting existing laboratory-based studies to remote paradigms means researchers are not reliant on subject pool databases and remote clinical studies can reach larger and more diverse samples and garner larger samples of special populations (4–7). Finally, allowing individuals to participate in studies from their homes has the potential to increase accessibility for those who might be physically, financially, or otherwise unable to come into a laboratory or clinic to participate.

There are, of course, pitfalls with remote assessments. Participants can take advantage of the format to bias their performance by recording responses and assessment materials manually or with screen capture software. Additionally, participants tested in their natural environments are vulnerable to external distractions otherwise not present in laboratory or clinic settings. Studies using “bring your own device” (BYOD) paradigms are additionally susceptible to distractions from device notifications unrelated to the experiment, and hardware and operating system (OS) differences across devices may influence behavioral data (8). In aging populations, perhaps the most critical consideration stems from the so-called “digital divide” which refers to demographic variables, such as age, gender, and socioeconomic status, that influence technology access and use behaviors (9). For example, in the context of aging research, the digital divide reflects the tendency for older adults to be less likely to use technology than younger adults and for older adults who use technology to do so less (10).

Due to technological advances and the popularity of smartphones, affordable smartphones are now widely available, and a greater number of older adults are adopting smartphones into their daily lives. The Pew Research Institute reported that 74% of Americans ages 50–64 and 42% of individuals 65 and older own a smartphone [a 16 and 12-percentage-point increase, respectively, compared with 2015; (11)]. However, these data were collected in 2017 and, critically, well before the COVID-19 pandemic, which has likely increased technology use among older adults. At our center, we have observed an increase in use of technology among our older adult research participants. From March 2020 to July 2021, use of videoconferencing software among our participants increased ~17%.

Despite increases in technology use, there remains considerable skepticism regarding older adults' ability to adapt to new technologies like smartphones and videoconferencing (12). If these perceptions are correct, then one would expect this digital divide to impact older adults' participation in smartphone-based remote studies. The present study aimed to address these longstanding age-related technology stereotypes by providing quantitative evidence that older adults—even those with little to no technology experience—are willing and capable of participating in intensive technology-enabled studies (i.e., longitudinal, multi-session remote studies). Specifically, we examined responses to a novel technology familiarity survey in conjunction with performance on an ongoing smartphone study of cognition.

## Methods

### Participants

Two samples of participants were recruited for the purposes of this study: The first sample included younger, middle-aged, and older adults recruited and tested online using Prolific (prolific.co; an online crowdsourcing portal for research studies) and compensated at a rate of $9.50/h for participation. Inclusion criteria for the Prolific studies included native English-speaking ability and United States as current country of residence. The second sample included participants recruited from ongoing studies of aging and dementia at the Charles F. and Joanne Knight Alzheimer's Disease Research Center (Knight ADRC) at the Washington University School of Medicine in Saint Louis. Specifically, the Healthy Aging and Senile Dementia (HASD) study was started in 1984 and is an ongoing study of the transition from cognitive normality to onset of dementia due to AD. All participants consented to participate in the study, and the Institutional Review Board approved all procedures at Washington University in St. Louis.

### Technology Familiarity

An assessment to measure age differences in technology acceptance, knowledge, familiarity, perceived difficulty, and usage was developed *de novo* for the purposes of this study. Although other instruments certainly exist to assess attitudes toward technology adoption [see work by Czaja et al. (13) amongst others], we aimed to additionally examine participants' objective technology knowledge in the present study. The theoretical foundation for this study used constructs from the Technology Acceptance Model (14, 15). Assessment constructs were focused on participant smartphone use behavior and ease of use. The assessment was administered using Qualtrics software and consisted of several sections that combined objective measurements and self-reported ratings. The objective items tested familiarity with multiple-choice questions regarding smartphone and social media icons. Participants were shown 17 icons (selected based on frequency of occurrence and centrality to common smartphone tasks) and asked to select what the icon represents from one of three options (the icons and survey can be viewed here: https://tinyurl.com/erh7s7j9). There were 11 smartphone-related icons (power, WiFi, location, delete, search, refresh, Bluetooth, airplane mode, settings, close, and share) and 7 software application-related icons (Instagram, Cash, Netflix, Facebook, Zoom, and Spotify) that participants were asked to identify in this section. Next, participants were asked if they owned a smartphone (defined for participants as “a mobile phone that has a touchscreen interface, internet access, and capable of running downloaded applications”). If they responded yes, they were asked a series of questions related to their smartphone usage. These questions included “How long have you owned a smartphone?”, “Does your phone plan include cellular data (i.e., you can access the Internet on the phone without needing a WiFi connection)?” and “How user-friendly do you find your smartphone? Think about things such as how intuitive the functions are and how easy it is to find what you are looking for.” Response options for these questions can be viewed in Table 1.

**Table 1:** Demographic data.

**Age group**	**Prolific 18–30**	**Prolific 31–55**	**Prolific 56 +**	**Knight ADRC 62–76**	**Knight ADRC 77+**	***p*-value^**b**^**
	***N =* 40^***a***^**	***N =* 41^***a***^**	***N =* 40^***a***^**	***N =* 113^***a***^**	***N =* 108^***a***^**	
**Age**	24.85 (3.95)	38.00 (6.14)	62.64 (5.18)	72.79 (2.76)	81.91 (4.15)	<0.001
**Gender**						0.044
Female	15 (38.0%)	16 (39.0%)	17 (44.0%)	54 (48.0%)	58 (54.0%)	
Male	22 (55.0%)	25 (61.0%)	22 (56.0%)	59 (52.0%)	50 (46.0%)	
Transgender female	1 (2.5%)	0 (0.0%)	0 (0.0%)	0 (0.0%)	0 (0.0%)	
Transgender male	1 (2.5%)	0 (0.0%)	0 (0.0%)	0 (0.0%)	0 (0.0%)	
Gender Non-conforming	1 (2.5%)	0 (0.0%)	0 (0.0%)	0 (0.0%)	0 (0.0%)	
**Race**						<0.001
Black or African American	5 (12.0%)	7 (17.0%)	2 (5.0%)	19 (16.8%)	9 (8.3%)	
White	30 (75.0%)	29 (71.0%)	38 (95.0%)	92 (81.4%)	99 (92.0%)	
Other^*c*^	5 (12.0%)	3 (7.3%)	0 (0.0%)	2 (1.8%)	0 (0.0%)	
Prefer not to answer	0 (0.0%)	2 (4.9%)	0 (0.0%)	0 (0.0%)	0 (0.0%)	
**Education**	14.72 (2.21)	16.40 (2.02)	15.72 (2.64)	16.70 (2.08)	16.19 (2.51)	<0.001
**APOE4 Status**						0.25
Negative				72 (65%)	78 (74%)	
Positive				38 (35%)	28 (26%)	
**Years of smartphone ownership**						0.32
<1 year	0 (0.0%)	3 (7.3%)	1 (2.6%)	5 (4.7%)	11 (11.0%)	
1–3 years	1 (2.5%)	1 (2.4%)	5 (13.0%)	12 (11.0%)	11 (11.0%)	
3–5 years	8 (20.0%)	1 (2.4%)	6 (15.0%)	14 (13.0%)	14 (12.0%)	
More than 5 years	31 (78.0%)	36 (88.0%)	27 (69.0%)	75 (71.0%)	63 (64.0%)	
**Has cellular data plan**						0.71
Yes	38 (97%)	39 (95.0%)	39 (100.0%)	95 (90.0%)	81 (82.0%)	
No	1 (2.6%)	2 (4.9%)	0 (0.0%)	3 (2.8%)	3 (3.0%)	
Not sure	0 (0.0%)	0 (0.0%)	0 (0.0%)	8 (8.2%)	15 (15.0%)	
**Smartphone user friendliness**
**(1** **=** **Very Easy; 5** **=** **Very Difficult)**	1.20 (0.46)	1.27 (0.55)	1.59 (0.68)	1.81 (0.96)	2.25 (1.04)	<0.001

a *Mean (SD); n (%)*.

b *Pearson's Chi-squared test; Omnibus Analysis of Variance*.

c *American Indian, Alaska Native, Native Hawaiian, Pacific Islander, or Asian*.

Next, participants were asked to rate the frequency with which they perform certain smartphone activities on a 6-point scale ranging from “Never” to “All the time.” Participants were asked to rate how often they performed the following 13 activities: “Make phone calls,” “Browse the web/Search for information,” “Use apps (for any purpose),” “Listen to music/podcasts/audiobooks/radio,” “Watch videos,” “Play games,” “Check the news,” “Take pictures,” “Record video,” “Check email,” “Text,” “Check social media,” and “Get directions/Use GPS.”

Finally, participants were asked to rate how easy or difficult it would be for them to perform 13 specific technology-related tasks using a 5-point scale ranging from “Extremely easy” to “Extremely difficult.” Participants were asked to assume that they were doing the task themselves with their current technology knowledge. Tasks included: “Touch-type on a keyboard (using both hands, not looking at keys),” “Search for information using a Web browser,” “Add an attachment to an email message,” “Set up a new modem/router in your home,” “Change your home WiFi password,” “Log in to a virtual private network (VPN),” “Download and install a new software program on a home computer,” “Download and install a new app on a personal smartphone,” “Use a self-checkout lane at a grocery store,” “Purchase items online,” “Log in to an online account using a public computer (no passwords saved),” “Change the privacy settings on a social media profile,” and “Set up automatic bill payments online.”

Several attention checks were included throughout the survey to ensure that participants read the instructions carefully and responded thoughtfully to the prompts (16). These included one in the technology task frequency rating section, which read “This is an attention check—please select ‘Never,”' and one in the difficulty rating section, which read “This is an attention check—please select 'Extremely difficult.” Participants were excluded if they failed to respond with the indicated answer to one of the two attention checks embedded in the rating sections.

### Ambulatory Research in Cognition (ARC) Study

In the present study, we focus on the enrollment rate, reasons participants elected not to participate, and study adherence rates in an ongoing smartphone-based study of cognition in normal aging and AD. The mobile assessment study from which the relevant metrics were drawn is called Ambulatory Research in Cognition (ARC) which uses a measurement “burst” design based on principles from ecological momentary assessment. Specifically, participants performed frequent but brief tests of episodic memory, attentional control, and processing speed administered at four pseudo-random times per day over seven consecutive days. Tasks were performed as participants went throughout their everyday lives (i.e., performing the tasks in familiar environments or wherever they were at the time of the notification). Participants received notifications before each 3–5 min assessment. Each session consisted of the three cognitive tasks (which tapped into the domains described above) and surveys regarding mood, sleep, fatigue level, and assessment context. Participants were reimbursed at a rate of $0.50 per completed assessment session. To incentivize participation consistency, participants receive bonus payments for completing all 4 sessions any given day ($1.00 per occurrence, max of $7.00), completing at least 2 assessments per day for 7 days ($6.00), and completing at least 21 assessments over 7 days ($5.00). The maximum compensation possible for one 7-day assessment visit was $32.00. Participant adherence is operationalized as the number of sessions completed throughout the 7-day visit period divided by 28 total assessment opportunities. The measures included in ARC have shown superior reliability and are correlated with age, in-clinic cognitive measures, and AD biomarkers (17), and have demonstrated sensitivity to time-of-day effects (18). Cross-sectional results of the cognitive measures, longitudinal performance, and relationships with AD biomarkers were not the focus of this report and will be disseminated elsewhere.

### Statistical Analyses

We assessed participants' technology knowledge, familiarity, and usage habits by comparing their responses to the survey questions using two-tailed *t*-tests, Pearson's correlations, and between-subjects analysis of variance (ANOVAs). We also adjusted for multiple comparisons using Bonferroni adjusted alphas^1^. Graphical representations and frequencies were used to describe the following: willingness to participate in ARC, individuals study refusal reasons, and adherence rates (i.e., adherence over seven consecutive days of cognitive assessments). Additionally, we examined the relationships between adherence and participant demographics and technology familiarity to directly test whether differences in race, education, gender, or technology familiarity/knowledge influence an individuals' ability to participate in a smartphone-based study.

## Results

Two cohorts of adults were administered a technology familiarity assessment, the first sample was recruited from Prolific (www.prolific.co), and the second sample was older adults from the Charles F. and Joanne Knight Alzheimer's Disease Research Center (Knight ADRC) who were enrolled in an ongoing smartphone-based study of cognition and risk of AD.

### Demographics

The Prolific sample included forty younger (aged 18–30), forty-one middle-aged (aged 31–55), and forty older adults (aged 56–76) who met inclusion criteria and were recruited to participate. One older adult failed to respond correctly to one or more attention checks that were embedded in the technology assessment to ensure adequate effort. Given the possibility that they either did not understand the instructions or were not engaged in the task, this older adult was removed before analysis. The Knight ADRC sample included 243 older adults (aged 66–93 years old). Seventeen participants were removed from analyses due to a Clinical Dementia Rating™ [CDR™; (19)] >0, indicating a clinical diagnosis of dementia. In addition, three participants were removed before analysis due to missing demographic data, and two were removed for failing the attention checks. Altogether, the analyses included 121 younger, middle-aged, and older adults from Prolific and 221 older adults from the Knight ADRC.

### Technology Familiarity

**Figure 1:**
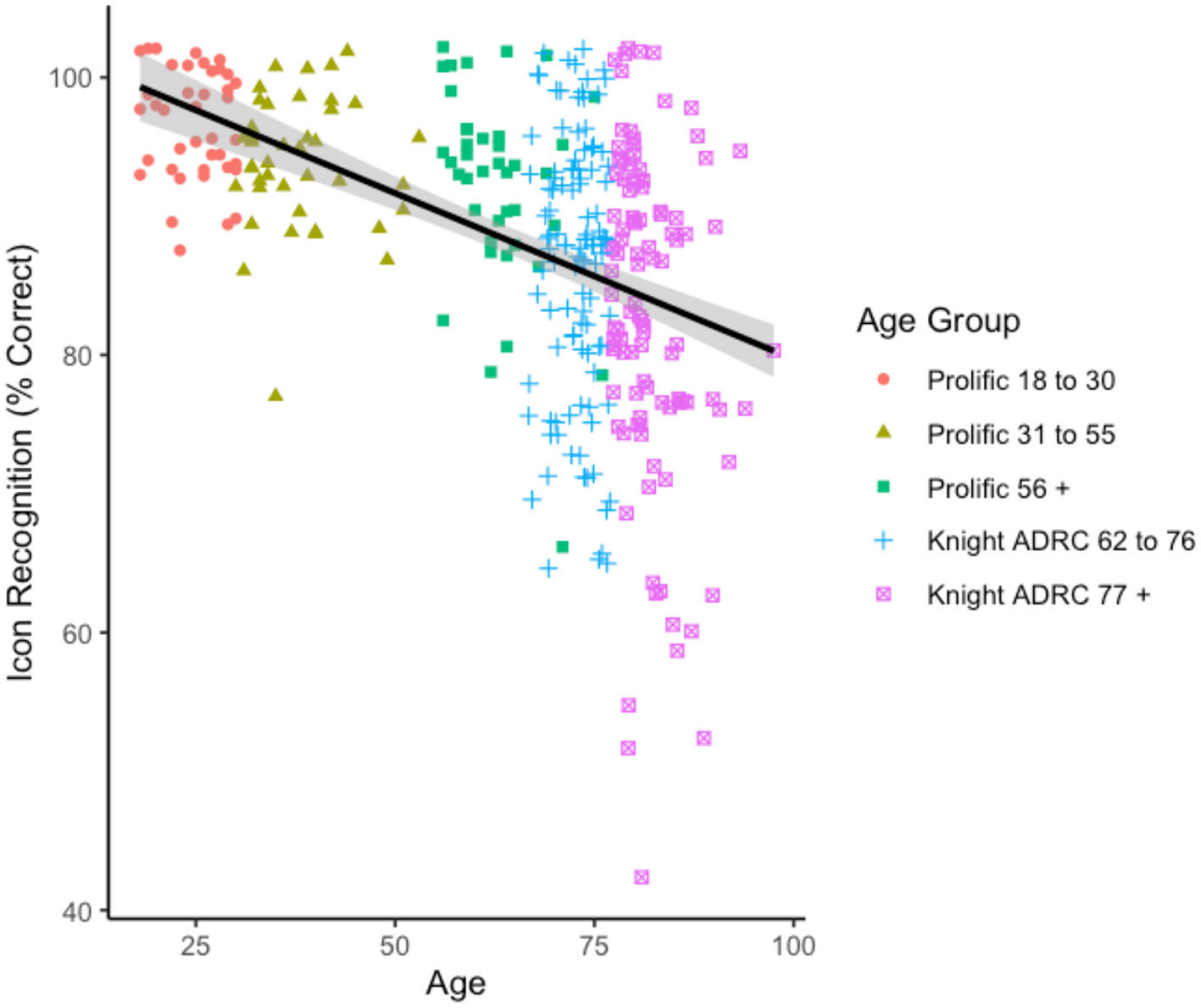
Icon recognition scores by age.

First, the relationship between age and recognition of technology-related icons was examined. Seven participants (four from the Knight ADRC 62–76 and three from the Knight ADRC 77 + group) were removed before analysis for having an icon knowledge score >3 standard deviations below the mean for their age group. As shown in Figure 1, there was a strong negative relationship between age and technology-related icon knowledge (i.e., percent correct), *r*(333) = −0.47, *CI*[−0.54, −0.38], *p* < 0.001^2^. There were no relationships between technology-related icon knowledge and either self-reported gender, race, or education over and above the effect of age, *p*s > 0.10.

Second, personal smartphone usage was examined. As shown in Table 1, there were no differences across age groups in the percentage of participants who reported owning a smartphone for more than 5 years [${?}_{\left(4,\text{N =}342\right)}^{2}$ = 4.68, *p* = 0.32] or having a cellular data plan, [${?}_{\left(4,\text{N}=342\right)}^{2}$ = 2.14, *p* = 0.71]. However, older participants reported finding their smartphones less user-friendly than younger participants, [*F*_(4, 318)_ = 15.85, *p* < 0.001]. Self-reported gender, race, and education were unrelated to these personal smartphone usage variables, *p*s > 0.12.

**Figure 2:**
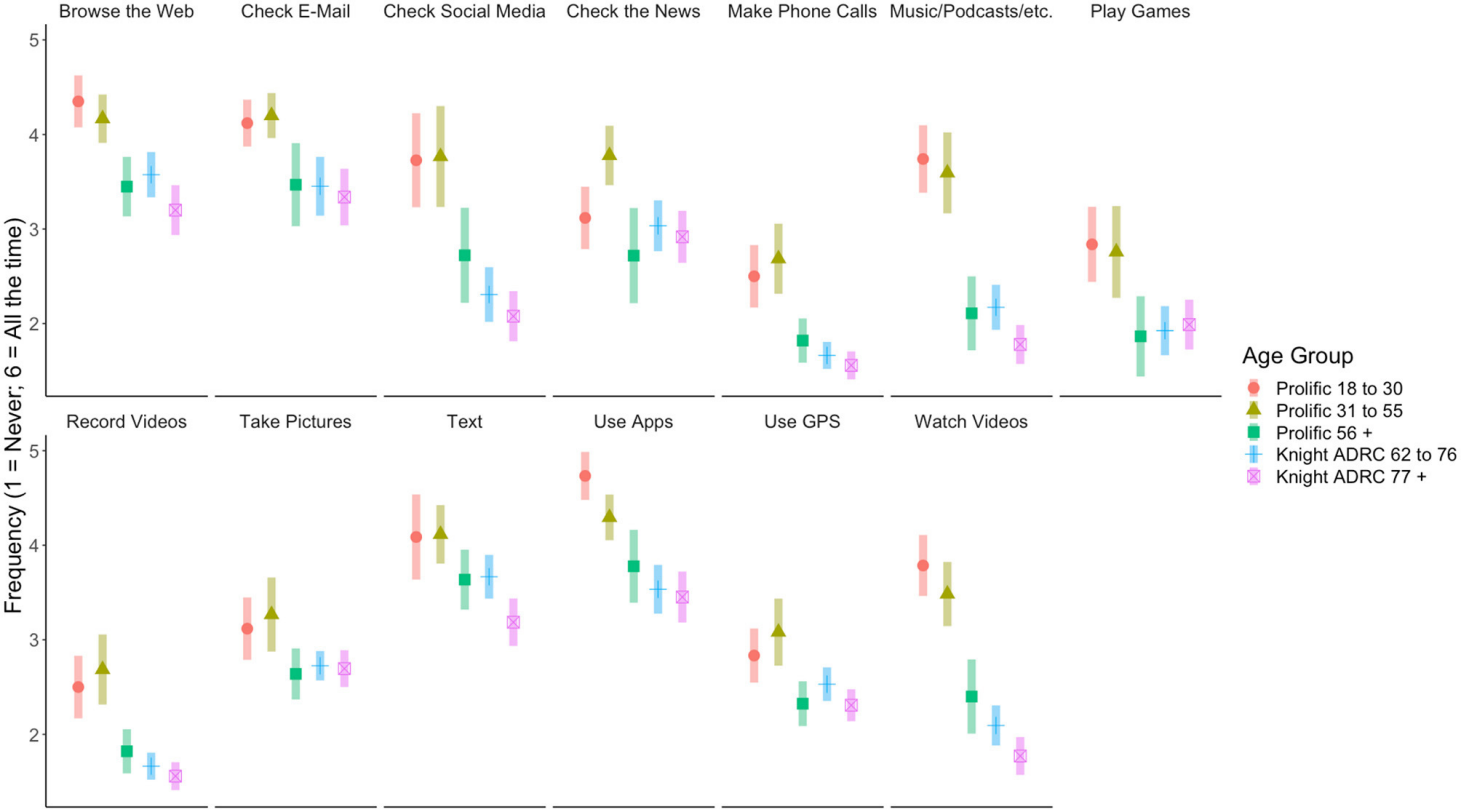
Participants' reported frequency performing technology-based tasks. Error bars represent 95% confidence interval.

As shown in Figure 2, there were omnibus age effects in self-reported frequency for all technology tasks assessed, *p*s < 0.002, suggesting a strong relationship between age and frequency of technology use. Interestingly, when modeled concurrently with age, there were no associations between frequency of technology use and self-reported gender, race, or education.

**Figure 3:**
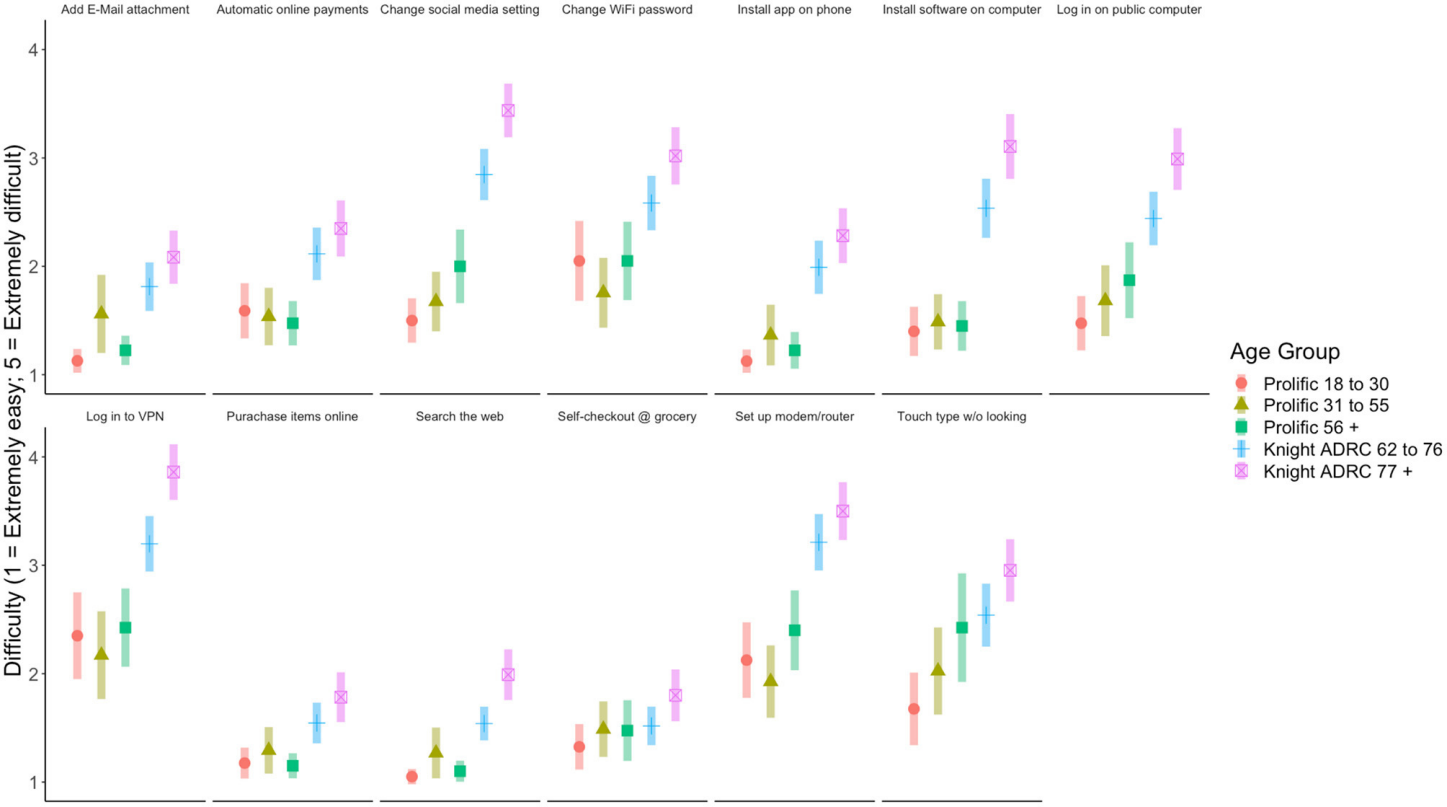
Participants' reported difficulty performing technology-based tasks. Error bars represent 95% confidence interval.

Difficulty ratings for performing everyday technology tasks are displayed in Figure 3. Similar to the frequency results, there were omnibus age group differences in difficulty ratings for all technology tasks assessed, *p*s < 0.02, except for “Use a self-checkout lane at a grocery store,” *p* = 0.06, which did not reach significance. There were no differences in participants' self-reported difficulty as a function of race. However, there were several differences as a function of gender and education, above and beyond the effect of age, that reached the corrected significance threshold. Specifically, females reported “Set up a new modem/router in your home” as being more difficult than males, *p* < 0.001. More years of education was associated with decreased difficulty ratings for the tasks “Search for information using a Web browser,” “Add an attachment to an email message,” “Download and install a new software program on a home computer,” “Download and install a new app on a personal smartphone,” “Purchase items online,” and “Log in to an online account using a public computer (no passwords saved),” *p*s < 0.002.

### Ambulatory Research in Cognition (ARC) Study Metrics

The focus of the present study was to investigate whether older adults are willing and able to participate in remote assessment studies on their smartphones, as such we report enrollment and refusal rates, the reasons participants chose not to participate, and adherence rates for an intensive smartphone-based remote cognitive assessment study at the Knight ADRC called Ambulatory Research in Cognition (ARC). The ARC study uses a measurement “burst” design that assesses cognition repeatedly with a maximum of 28, 3-min testing sessions spread over a week.

**Figure 4:**
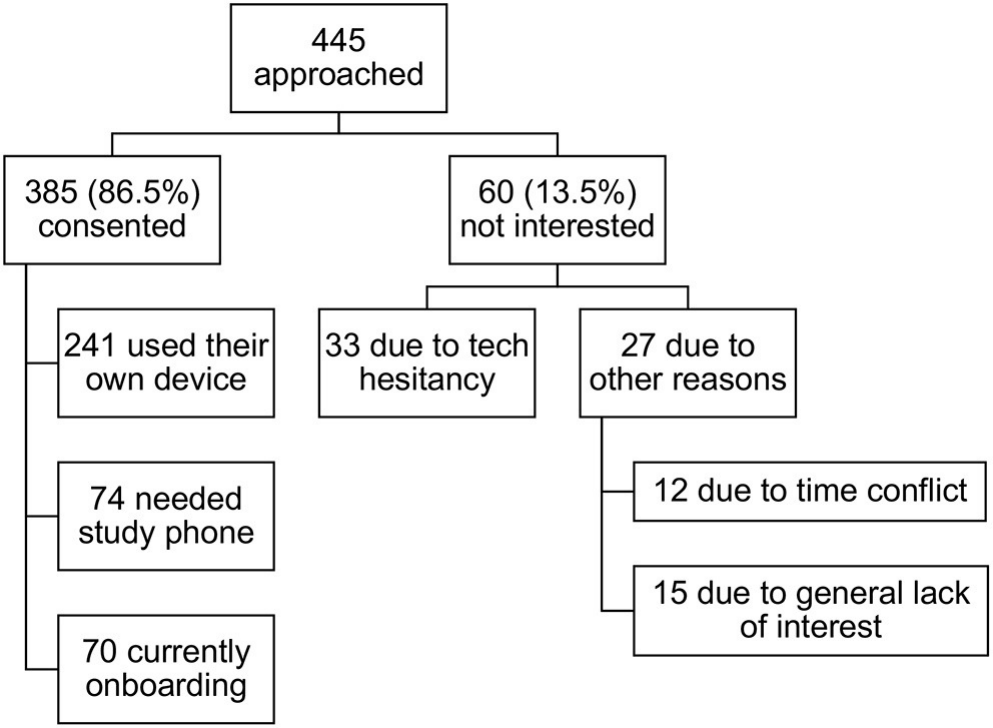
ARC study enrollment rates.

As shown in Figure 4, 445 individuals in the Knight ADRC cohort were approached with the opportunity to participate in the ARC study. Of the individuals approached, 86.5% consented to participate. For the 13.5% who were not interested in participating, we examined whether these individuals chose not to participate because they were hesitant to partake in a study requiring smartphone use or if they chose not to participate due to non-technology-related reasons (e.g., time constraints, general lack of interest in participation, etc.). Of the 60 individuals who elected not to participate in the study, 33 mentioned technology (specifically, smartphone) hesitancy. The remaining 27 indicated non-technology-related reasons (see Figure 4 for a more detailed breakdown).

We were also interested in whether demographic characteristics such as self-reported gender, race, or education may have been a factor in participation. As shown in Table 2, the demographics of the individuals who declined to participate in ARC closely resembled those who chose to enroll (see Table 1). This suggests that neither self-reported gender, race, nor education appeared to influence whether or not an individual decided to participate in ARC.

**Table 2:** Individuals who declined ARC participation.

**Characteristic**	***N =* 60^*a*^**	**vs. ARC participants^***b***^**
**Age**	81.34 (6.43)	0.54
**Gender**		0.56
Female	34.0 (57.6%)	
Male	25.0 (42.4%)	
Unknown	1	
**Race**		0.97
Black or African American	5.0 (8.5%)	
White	54.0 (91.5%)	
Unknown	1	
**Education**	15.98 (2.36)	0.60
**APOE4 status**		0.83
Negative	40.0 (67.8%)	
Positive	19.0 (32.2%)	
Unknown	1	

a *Mean (SD); n (%)*.

b *t-test/Chi-squared test p-value*.

**Figure 5:**
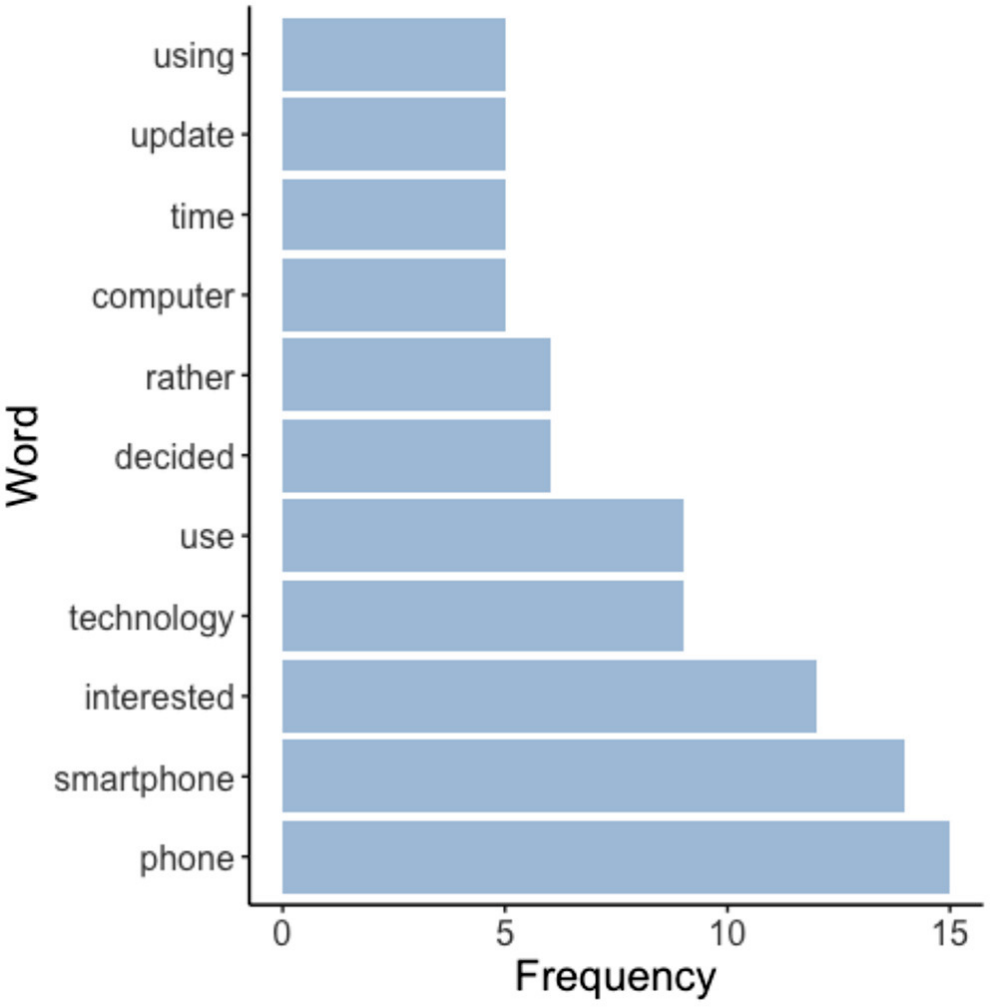
Words used by individuals who declined to participate in the mobile assessment study.

Next, the frequency of specific words mentioned in participants' refusal reasons (recorded manually at the time of consent) is shown in Figure 5. Consistent with the refusal reason breakdown reported above, the words “phone,” “smartphone,” and “technology” were amongst the most common words used in the explanations participants gave for not wanting to participate—indicating that technology hesitancy was indeed a deterrent for the small proportion of older adults' who were approached but chose not to participate in ARC.

**Figure 6:**
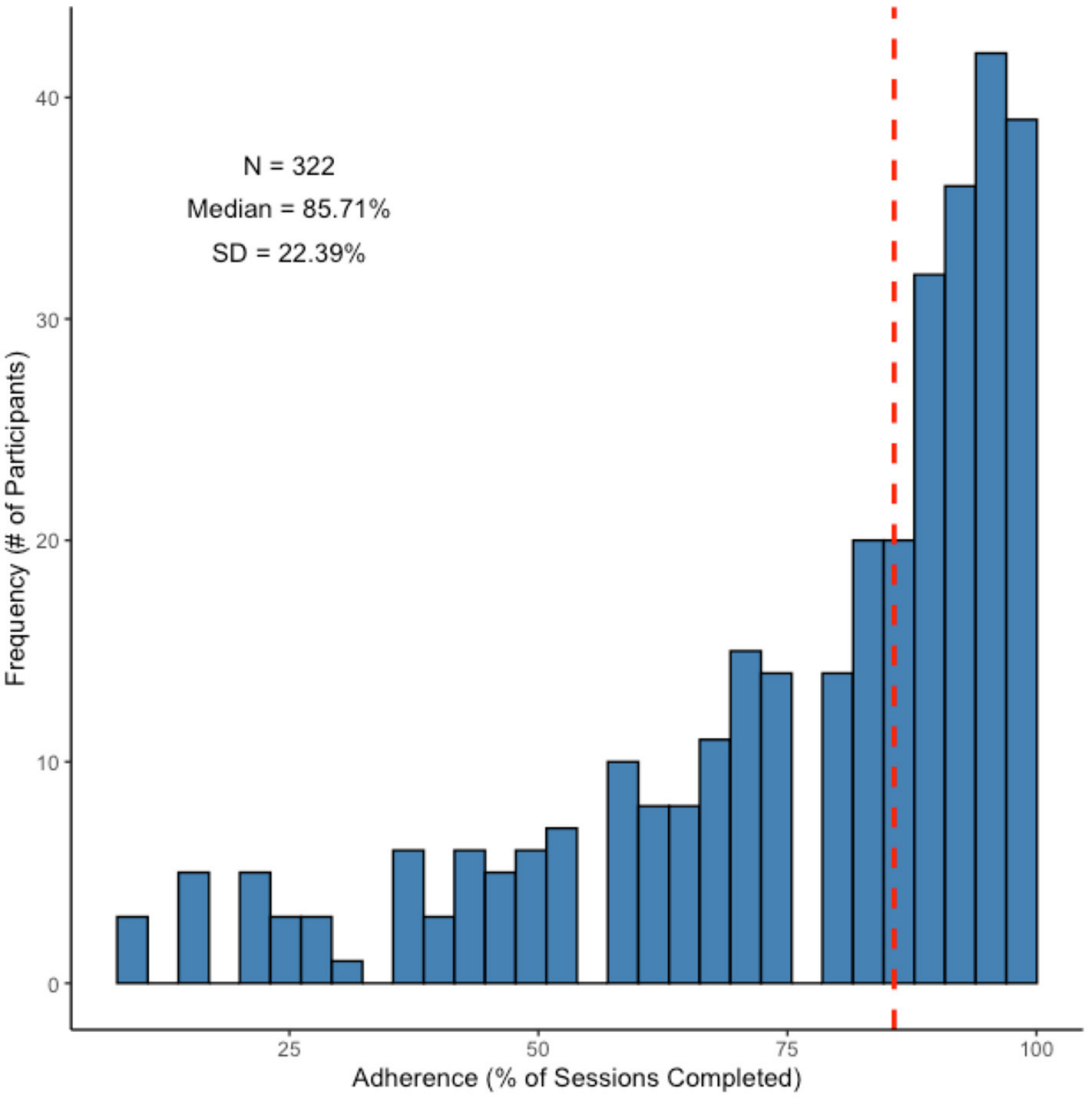
Histogram of adherence rates for participants enrolled in the mobile assessment study. Dashed (red) line indicates the median adherence.

**Figure 7:**
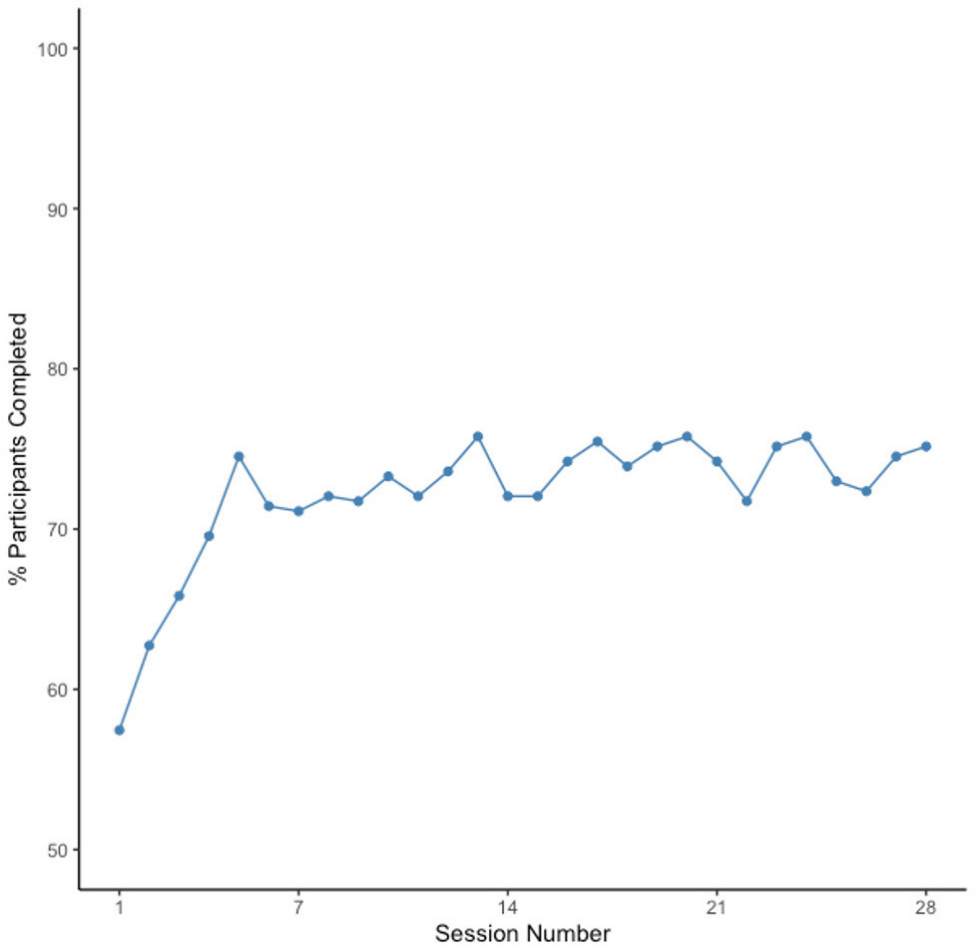
Percentage of participants who completed each of the 28 ARC sessions.

As shown in Figures 6, 7, not only were older adults quite willing (86.5% enrollment rate) to participate in the mobile assessment study, they also did exceedingly well at completing the 28 repeated testing sessions. The median adherence rate, defined as the percentage of sessions that participants completed, was 85.71%, with adherence rates ranging from 11 to 100%. The modal adherence rate was 96.43% with the distribution of adherence rates being highly left-skewed (see Figure 6).

Finally, we examined whether any relationships existed between adherence and participant demographics or technology familiarity. Specifically, we tested whether gender, race, education, or technology familiarity/knowledge influenced ARC adherence. Although age was negatively correlated with adherence, *r* = −0.18, *p* < 0.001, neither technology task frequency ratings, *p*s > 0.10, nor perceived technology task difficulty ratings, *p*s > 0.21, were associated with adherence. Furthermore, adherence did not differ as a function of self-reported gender, *p* = 0.27, race, *p* = 0.89, years of education, *p* = 0.20, nor technology icon recognition accuracy, *p* = 0.10.

## Discussion

We investigated age differences in technology knowledge, familiarity, and usage and examined the willingness of a clinical sample of older adults to enroll and to adhere to an intensive smartphone-based remote cognitive assessment. First, we compared younger, middle-aged, and older adults on technology familiarity, knowledge, perceived difficulty, and usage habits using a novel technology familiarity assessment. Second, we examined participation in the smartphone-based ARC study focusing on enrollment rates, refusal factors, and adherence rates. Finally, we investigated whether technology familiarity and interactions with demographic characteristics, including gender, race, and education, influenced participation in the smartphone study.

Regarding age differences in technology familiarity, we found robust, negative associations between age and technology familiarity. As expected, older age was associated with less technology knowledge (icon recognition accuracy), lower smartphone user-friendliness ratings, less frequency of technology use, and more difficulty performing everyday technology tasks. However, despite these apparent age differences in technology knowledge and familiarity, the majority (86.5%) of older adults approached about participating in the intensive smartphone-based study of cognition at the Knight ADRC elected to participate and showed exceedingly good adherence (85.71% of sessions completed). Indeed, these rates are comparable to those of highest performer groups demonstrated in a recent cross-study evaluation of 100,000 participants (20). Of the individuals who declined to participate, ~50% cited technology hesitancy as the reason they did not want to participate in the study. However, among those enrolled, neither technology familiarity, knowledge, perceived difficulty, nor self-identified gender, race, or education was associated with participants' adherence. Ultimately, we argue that the notion that older adults are uninterested and/or unable to participate in smartphone-based remote assessment studies is based on biases such as ageism, not empirical data. Further, the present findings do not support the claim that self-reported gender, race, or level of education make an individual more or less willing or able to participate in remote smartphone-based studies.

In addition to these findings, several important broader implications can be drawn from this investigation. The high enrollment and adherence rates for the ongoing ARC smartphone study provide evidence that researchers can ameliorate accessibility limitations and technology hesitancy among older adults with cost-effective strategies that encourage and facilitate participation. For example, as done for several individuals enrolled in ARC (see Figure 4), investigators can provide participants who cannot afford, or who do not otherwise have access to, a study-eligible smartphone with a readily affordable model that meets requirements for study participation. Additionally, our ARC study team found that informing participants that onboarding (i.e., app download, set up, walk-through, etc.) is done alongside study personnel (either in person or via videoconference) reduced hesitancy and concerns about overcoming technology-related challenges unassisted^3^. Moving forward, although further research is certainly warranted, smartphone-based remote assessments may offer a more practical and logistically plausible solution for large-scale studies. Allowing individuals to participate in research studies and health monitoring assessments on their own schedules and in their natural environments may increase engagement, sample size, and diversity, and make participation more accessible for many who may otherwise be unable or unwilling to come to the lab or clinic for in-person studies.

The findings of this study should be considered in light of broader considerations and limitations which may be addressed in future studies. First, as mentioned in the introduction, the present study's focus was to examine the claim that older adults' lack of technology familiarity may inhibit their willingness/ability to participate in smartphone-based studies. ARC test performance as a function of age and CDR status, reliability, and correspondence with in-lab measures will be explored in future studies. Second, it is important to note that because we used an online recruitment platform (i.e., Prolific), the older adults recruited from Prolific may be more technologically savvy than older adults in the general population (see text footnote 1). We included this participant sample because they provide an important comparison group comprised of more technologically familiar older adults who frequently participate in online studies, as opposed to the Knight ADRC participants that were recruited through a more traditional clinical setting. The technology familiarity of the general population of older adults most likely lies somewhere in between these two groups. Third, as mentioned in the Introduction, BYOD designs are susceptible to hardware and OS differences across devices—although this issue was not directly investigated here, studies in our lab (Nicosia et al., in prep.) and others (8) are well underway as the literature on BYOD remote assessments continues to grow. Finally, the Knight ADRC participants are a clinical research sample that consist of highly educated and primarily White older adults motivated to engage in extensive imaging and fluid biomarker studies. This may have impacted their ARC adherence and performance and therefore limit the generalizability of the findings presented here. However, with these important limitations considered, it is notable that the participant sample demonstrated expected age-related trends regarding technology adoption and demonstrably less technology familiarity, yet were clearly capable of participating in an intensive smartphone-based cognitive assessment regardless of gender, race, or education level. Thus, despite potential limitations of the current sample, the present study demonstrates that ARC, and other remote assessment techniques, has the potential to reach larger and more diverse samples in future studies.

Our findings suggest that the majority of older adults who were approached elected to participate in the ARC study and showed exceptional adherence despite the influence of age on technology familiarity. Furthermore, neither technology familiarity, knowledge, perceived difficulty, nor self-identified gender, race, or education were associated with participants' adherence. Ultimately, these results suggest that videoconference, online, and smartphone-based remote assessments may offer more accessible, inclusive, and logistically plausible solutions for large-scale clinical studies with older adults.

## Data Availability Statement

The raw data supporting the conclusions of this article will be made available by the authors, without undue reservation.

## Ethics Statement

The studies involving human participants were reviewed and approved by Washington University Human Research Protections Office. The patients/participants provided their written informed consent to participate in this study.

## Author Contributions

JH, SA, AA, and JN contributed to conception and design of the study. SS, SA, MT, and JN coordinated data collection. JN performed the statistical analysis, which were verified by JH. JN wrote the first draft of the manuscript. JH and JB-B wrote sections of the manuscript. JH and JM acquired funding for the study. All authors contributed to manuscript revision, read, and approved the submitted version.

## Funding

This work was supported by NIA Grants P30AG066444, P01AG03991, and P01AG026276 (PI JM) and R01AG057840 (PI JH) and a grant from the BrightFocus Foundation A2018202S (PI JH). We would also like to thank the Shepard Family Foundation for their financial support.

## Conflict of Interest

The authors declare that the research was conducted in the absence of any commercial or financial relationships that could be construed as a potential conflict of interest.

## Publisher's Note

All claims expressed in this article are solely those of the authors and do not necessarily represent those of their affiliated organizations, or those of the publisher, the editors and the reviewers. Any product that may be evaluated in this article, or claim that may be made by its manufacturer, is not guaranteed or endorsed by the publisher.

## Footnotes

^1^Specifically, alphas from the ANOVAs examining participants' self-reported frequency and difficulty ratings were corrected for multiple comparisons. This resulted in a corrected alpha of 0.0038 (i.e., 0.05/13 = 0.0038).

^2^Follow-up Bonferroni-adjusted pairwise comparisons indicated that, there were no age group differences across the Prolific samples, *p*s > 0.16. The Knight ADRC 62 to 76 group showed less icon knowledge than all groups (except for the Knight ADRC 77 + group), *p*s < 0.04 and the Knight ADRC 77 + group had lower icon knowledge scores as compared to all groups, *p*s < 0.04. Overall, the Prolific participants showed better icon knowledge than the Knight ADRC participants, *p* < 0.001.

^3^The specific protocol and scripts used by our study team to support engagement can be provided to researchers wishing to replicate ARC procedures upon request. For additional information on factors associated with increased participant engagement, see Pratap et al. (20).
